# Dichroic spin–valley photocurrent in monolayer molybdenum disulphide

**DOI:** 10.1038/ncomms8636

**Published:** 2015-07-02

**Authors:** Mustafa Eginligil, Bingchen Cao, Zilong Wang, Xiaonan Shen, Chunxiao Cong, Jingzhi Shang, Cesare Soci, Ting Yu

**Affiliations:** 1 School of Physical and Mathematical Sciences, Physics and Applied Physics, Nanyang Technological University, 21 Nanyang Link, Singapore 637371, Singapore; 2 Centre for Disruptive Photonic Technologies, Nanyang Technological University, 21 Nanyang Link, Singapore 637371, Singapore; 3 Department of Physics, Faculty of Science, National University of Singapore, 2 Science Drive 3, Singapore 117551, Singapore; 4 Centre for Advanced 2D Materials and Graphene Research Centre, National University of Singapore, 6 Science Drive 2, Singapore 117546, Singapore

## Abstract

The aim of valleytronics is to exploit confinement of charge carriers in local valleys of the energy bands of semiconductors as an additional degree of freedom in optoelectronic devices. Thanks to strong direct excitonic transitions in spin-coupled *K* valleys, monolayer molybdenum disulphide is a rapidly emerging valleytronic material, with high valley polarization in photoluminescence. Here we elucidate the excitonic physics of this material by light helicity-dependent photocurrent studies of phototransistors. We demonstrate that large photocurrent dichroism (up to 60%) can also be achieved in high-quality molybdenum disulphide monolayers grown by chemical vapour deposition, due to the circular photogalvanic effect on resonant excitations. This opens up new opportunities for valleytonic applications in which selective control of spin–valley-coupled photocurrents can be used to implement polarization-sensitive light-detection schemes or integrated spintronic devices, as well as biochemical sensors operating at visible frequencies.

## Abstract (web summary)

Valleytronic materials allow for band structure minima to be exploited in electronic transport devices in addition to charge and spin. Here, the authors demonstrate selective control of spin–valley-coupled photocurrents via circularly polarized light in molybdenum disulphide grown by chemical vapour deposition.

## Graphical abstract

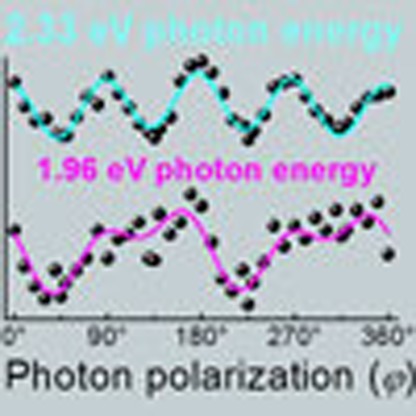

Research on two-dimensional (2D) materials synthesis and characterization has accelerated since the discovery of graphene^1^, for the purpose of utilizing these multifunctional materials in highly efficient nano- and biotechnological platforms^2^. Among 2D materials, particularly transition metal dichalcogenide (TMD) monolayers^3^,^4^ have shown various interesting properties such as charge density waves^5^, unusual strain^6^ and thermal energy dependence^7^, tunable light emission by chemical control^8^ and fast photoresponse^9^, which can be used in interdisciplinary device applications. Today's nanodevices mostly use charge and spin degree of freedom; however, it is also possible to exploit the valley degree of freedom, which is simply the confinement of charge carriers in momentum space of 2D materials. For instance, in molybdenum disulphide (MoS_2_)—a 2D semiconductor TMD and a non-centrosymmertic crystal—inherent broken inversion symmetry in monolayers leads to a large spin–orbit interaction (SOI) that splits the valence band (VB) by 150 meV^10^ and gives rise to strong excitonic transitions due to the direct band gap at low energy *K* and *−K* valleys. The upper (lower) VB is associated with the *A* (*B*) excitons. The same broken inversion symmetry together with time reversal symmetry is responsible for spin–valley coupling in monolayer MoS_2_ and similar TMDs (WS_2_, WSe_2_ and MoSe_2_); that is, the sign of the hole spin in the upper (or lower) VB will oppose in different valleys. The descriptive Hamiltonian^10^ can be simply expressed as 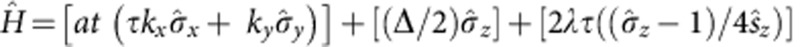 where *a* is the lattice constant, *t* the effective hopping integral, *τ* (±1) the valley index, 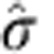 the Pauli matrices, Δ the energy gap, 2*λ* the spin splitting at the VB, and 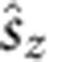 the Pauli matrix for spin; *k*_*x*_, *k*_*y*_, *k*_*z*_ are the momentum components in *x*, *y*, *z* coordinates, respectively. The first term is related to the valley related hopping, the second term is purely spin dependent and the last term describes the spin-dependent phenomena as a result of SOI in each valleys. The spin–valley coupling in the last term, which leads to the valley polarization, was demonstrated experimentally by light helicity-dependent photoluminescence measurements corresponding to the *A* and *B* excitons in monolayer MoS_2_^11^,^12^. Recently, in suspended monolayer MoS_2_ samples, photocurrent (PC) due to the *A* and *B* excitons was also revealed^13^. A useful framework to study light–matter interactions in 2D materials is based on PC experiments performed by circularly polarized excitation^14^. However, helicity-dependent PCs due to excitons with on-resonance and off-resonance excitations in monolayer TMDs are still to be uncovered.

There have been extensive studies on growth techniques of TMDs to synthesize high-quality, single-crystal and large-area samples. Among these efforts, high-quality single layer but small area (<10 μm) samples that are mechanically cleaved, have shown strong photoluminescence, fluorescence^15^,^16^, high-mobility–low-power switching^17^, and large photoresponse^18^. Alternatively, samples grown by chemical vapour deposition (CVD) methods^19^,^20^,^21^,^22^ also yield quite intense light emission^23^, particularly monolayers of tungsten disulphide (WS_2_) have shown stronger photoluminescence than mechanically cleaved monolayers^24^. In terms of electrical transport, both mechanically cleaved and CVD grown WS_2_ and MoS_2_ have *n*-type behaviour. In addition, since CVD method allows also growing multiple 2D materials to form hetero- and hybrid structures^4^, it offers to tailor functional physical systems and, particularly for optoelectronics and spin-dependent valleytronics. The possibility of synthesizing high-quality samples with much larger size (>500 μm) than mechanically cleaved samples gives CVD growth techniques a major advantage in the development of future technologies based on TMDs.

In this work, we demonstrate spin-coupled valley-dependent dichroic PC of a CVD grown single-layer MoS_2_ phototransistor excited by on-resonance and off-resonance photon energies (1.96 and 2.33 eV, respectively). A PC due to the circular photogalvanic effect (CPGE) arises as a result of circularly polarized light incident on monolayer MoS_2_ with an oblique angle. The spin–valley coupling, the valley selection rules and the excitation frequencies determine the magnitude of PC for left and right circularly polarized states. For excitations that are on-resonance with the excitons, there should be a difference between the CPGE PCs due to left and right circularly polarized excitations; while for excitations, which are off-resonance with the excitons, there should be no difference between these CPGE PCs. Our PC measurements evidence this variation and reveal a high-CPGE PC polarization. We remark on further possibilities of making use of this unique opto-valleytronic control for interdisciplinary applications.

## Results

### Spin–valley coupling and circular photogalvanic effect

The phenomenological expression of light helicity-dependent PC is strictly determined by the crystal symmetry, the angle of incidence, *θ*, (with respect to the normal to the sample plane), the azimuthal angle, *φ*, (light propagation with respect to the *x*-direction in the *x*–*y* plane) of the excitation beam and the angle of photon polarization, *ϕ*, (with respect to the electric field vector direction)^14^. In transverse geometry (as shown in the experimental configuration) in which light is directed in the *x*–*z* plane and the PC is measured in the *y*-direction, *φ* is fixed. If not otherwise specified, in this work, we used *φ*=90° and *θ*=45°, so that by rotating a quarter-wave plate (QWP), polarization of a linearly polarized laser can be varied from 0° linear (↔) to 45° left circular (σ−), back to 90° linear (↔) to 135° right circular (σ+) and then back to 180° linear (↔) polarization, and so on.

Monolayer MoS_2_ has 
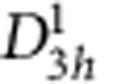
 crystal symmetry^10^,^11^,^12^. When a Mo atom is in an inversion centre, there is no coinciding atom when a S atom is projected to the 2D plane. When such a symmetry system is subjected to a circularly polarized light field with an oblique angle *θ*, (*θ*≠0°) a PC with sin 2*ϕ* dependence—current due to the CPGE, *j*_CPGE_ will be the major PC. The angle of incidence *θ* dependence of *j*_CPGE_ is sin *θ*. At the same time, a PC with cos 4*ϕ* dependence, which is due to the transfer of light momentum to electrons, is also present—the so-called linear photon drag effect (LPDE), *j*_LPDE_^14^. In addition, a PC similar to *j*_CPGE_ in origin, but related to the linear polarization of light as *j*_LPDE_—linear photogalvanic effect (LPGE), *j*_LPGE_^14^ is also possible, although it should ideally vanish at *σ*+ and *σ*− excitations. On the basis of these three contributions, the total PC can be generally expressed by the phenomenological formula *J*=*C*_1_ sin 2*ϕ*+*L*_1_ sin 4*ϕ*+*L*_2_ cos 4*ϕ*+*D*. Here *C*_1_ is the coefficient for the CPGE current (*j*_CPGE_=*C*_1_ sin 2*ϕ*); *L*_1_ for the LPGE current (*j*_LPGE_=*L*_1_ sin 4*ϕ*); *L*_2_ for the LPDE current (*j*_LPDE_=*L*_2_ cos 4*ϕ*); and *D* is polarization-independent term.

**Figure 1:**
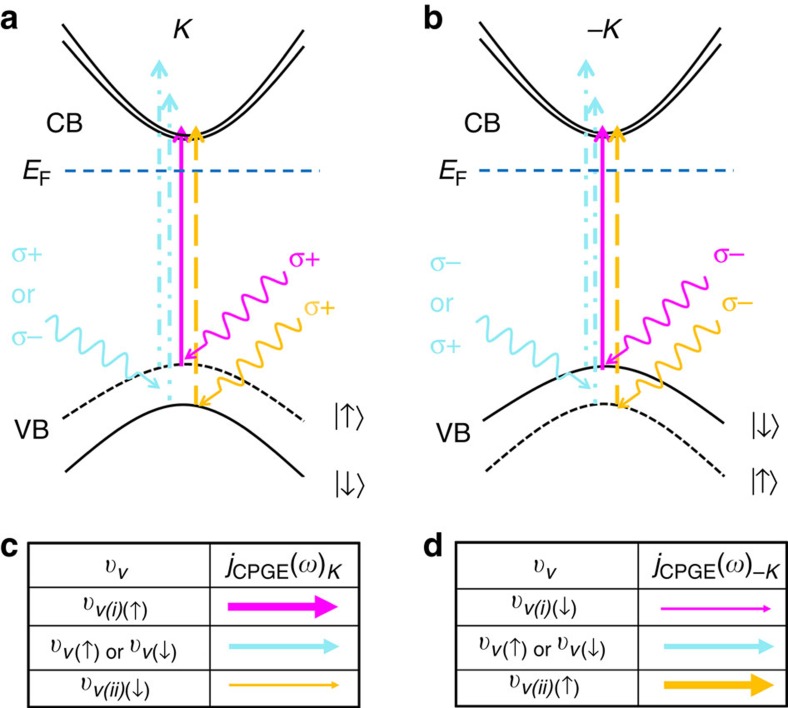
Band diagram of *K* and –*K* valleys of MoS_2_ and photocurrent generation. The *σ*+ and *σ*− excitations which are on-resonance with *A* exciton—a hole in the upper valence band (VB) (black solid (dash) curve in **a**.**b** for *K* (−*K*) valley) and electron in the conduction band (CB) (black solid curves in **a**,**b**) lead to photocurrent (PC) generation in *K* and −*K* valleys, respectively. Similarly, the *σ*+ and *σ*− excitations which are on-resonance with *B* exciton—a hole in the lower VB (black solid (dash) curve in **a**,**b** for *K* (−*K*) valley) and electron in the CB lead to PC generation. The blue horizontal dashed lines in **a**,**b** are the Fermi level E_F_ which can be tuned by *V*_g_. The VBs are split by 150 meV, while the splitting in the CB is negligible. The purple vertical solid (orange long dash) arrows show interband transitions associated with the *A* (*B*) excitons when illuminated by on-resonance laser; while turquoise vertical dash-dot arrows show possible transitions by off-resonance laser. For on-resonance laser, *σ*+ (*σ*−) excitation in *K* (−*K*) valley is linked with hole spin ↑ (↓) due to the spin–valley coupling for *A* excitons. Thus based on theory, the circular photogalvanic current in *K* valley, *j*_CPGE_(*ω*)_*K*_ due to the *υ*_*v*(*i*)**↑**_ at *σ*+ excitation will be larger (shown by thicker purple right arrow in **c** as a result of transitions represented by purple vertical solid arrows in **a**) than the *j*_CPGE_(*ω*)_−*K*_ due to the *υ*_*v(i)*↓_ at *σ*− excitation (shown by thinner purple right arrow in **d** as a result of transitions represented by purple vertical solid arrows in **b**), which is the reason of the pronounced photocurrent polarization. Similar but opposite scenario for *υ*_*v*(*ii*)↓_ and *υ*_*v*(*ii*)**↑**_, by exchanging valleys and spins applies for *B* excitons (orange right arrows). For off-resonance laser, *σ*+ and *σ*− excitations yield a negligible photocurrent polarization due to simultaneous populations of both valleys, as shown by equivalent turquoise right arrows in both **c**,**d**.

In a recent work on multilayer WSe_2_, the *j*_CPGE_ was observed due to absorption at Λ valleys—intraband transitions within the conduction band (CB)^25^. In the case of monolayer MoS_2_, spin–valley coupling will be at *K* valleys and the *j*_CPGE_ should be due to interband transitions from the VB to the CB, for circularly polarized radiation with energies closer to the exciton energies. When spin couples to valley^10^, sign of the spin states of *A* (or *B*) excitons in one valley oppose to that in the other valley. By a simple approach, when excited by *σ*+ (*σ*−) excitations with energy slightly higher than the formation/dissociation energy of *A* exciton, an electron in VB makes an interband transition in *K* (*−K*) valley, with hole spin up ↑ (spin down ↓). Similarly, when excited by *σ*+ (*σ*−) excitations with energy slightly higher than the formation/dissociation energy of *B* exciton, an electron in VB makes an interband transition in *K* (*−K*) valley, with hole ↓ (↑) (Fig. 1). This coupling can be understood by the PC generated when circularly polarized light-field irradiated on the sample is perpendicular to the electrodes (as shown in the experimental setup). In such a case, the velocities **υ**_***v***_ of the carriers in the VB, which absorb this light field, will have a contribution additional to the one due to the band dispersion δ*ɛ*(**k**)/δ**k** for energy *ɛ*(**k**) in **k** space. That contribution is the anomalous velocity which appears as the second term in ***υ***_**v**_(**k**)=(1*/ħ*)(δ*ɛ*(**k**)/δ**k**)—((*e/ħ)***E** × **Ω**)^26^, where *ħ* is the Planck constant, **E** the electric field vector, and **Ω** the Berry curvature that has the same magnitude but different sign in *K* and −*K* valleys, 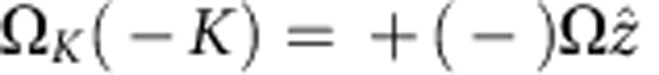. Then, for excitation that is on-resonance with the *A* excitons, we can write the magnitude of **υ**_**v**_(**k**) at *K*(−*K*), which actually depends on the light helicity, as *υ*_*v(i)***↑**(↓)_=*|*(*e/ħ*)**E** × **Ω**_*K*(–*K*)_|; and for excitation which is on-resonance with the *B* excitons, as *υ*_*v(ii)*↓(**↑**)_=*|*(*e/ħ*)**E** × **Ω**_*K*(−*K*)_|. In this case, the PC due to the *υ*_*v(i)***↑**_ (*υ*_*v(ii)*↓_), at *σ*+ excitation will be larger than the PC due to the υ_*v(i)*↓_ (*υ*_*v(ii)***↑**_) at *σ*− excitation. As an example, for the *A* excitons, the CPGE current *j*_CPGE_ (*ħω*), (*ω* is the excitation frequency) is given by *j*_CPGE_(*ω*)_σ+ (σ−)_ or *j*_CPGE_*(ω)*_*K*(−*K*)_=(8*eπ*/*ħ*) [∑_*v*(*i*)→*c*_ (*υ*_*c*_*τ*(*ɛ*_*c*_)—((δ*ɛ*_*v(i)*_/δ*k*)—*υ*_*v*(*i*)**↑**(↓)_*τ*(*ɛ*_*v*(*i*)_))] |*M*_*v*(*i*)→*c*_| |*f*(*ɛ*_*c*_)–*f*(*ɛ*_*v(i)*_)| *δ* (*ɛ*_*c*_*–ɛ*_*v(i)*_–*ħω*), and (*υ*_*c*_*τ*(*ɛ*_*c*_))>((δ*ɛ*_*v(i)*_/δ*k)*—*υ*_*v(i)***↑**(↓)_*τ*(*ɛ*_*v(i)*_)); where *v*(*i*) is the initial VB state on-resonance with the *A* excitons and *c* is final CB states, *M*_*v*→*c*_ is the transition matrix from the upper VB states to CB states, *υ*_*c*_ is the electron velocity in the CB states, *τ*(*ɛ*_*v*_) and τ(*ɛ*_*c*_) are the momentum relaxation times in the VB and CB, respectively. The Fermi–Dirac functions *f*(*ɛ*_*c*_) and *f*(*ɛ*_*v*_) are dependent on the gate voltage and to study purely interband transitions, the Fermi level must be lowered by negative gate voltage (off state). Here we remark at the condition (*υ*_*c*_*τ*(*ɛ*_*c*_))>((δɛ_v_/δ*k*)—*υ*_*v***↑**(↓)_*τ*(*ɛ*_*v*_)), which determines the sign of *j*_CPGE_(*ω*)_*K*(−*K*)_. We can consider the *j*_CPGE_(*ω*)_*K*(−*K*)_ with three components: (1) ∝ *υ*_*c*_*τ*(*ɛ*_*c*_); (2) ∝ (δ*ɛ*_*v*_*/*δ*k*); and (3) ∝ *υ*_*v*(*i*)**↑**(↓)_*τ*(*ɛ*_*v*(*i*)_). The third part (anomalous part) is the one that differs for on-resonance excitations. Anomalous parts of the electron velocities in the upper VB states for *K* and −*K* valleys for excitations on-resonance with the *A* excitons are *υ*_*v*(*i*)**↑**_ and *υ*_*v*(*i*)↓_, respectively, which are equal in magnitude but opposite in sign. For excitations on-resonance with the *B* excitons, anomalous parts of the electron velocities in the lower VB states for *K* and −*K* valleys are υ_*v*(*ii*)**↑**_ and υ_*v*(*ii*)↓_, respectively. On the basis of valley selection rules, the sign of *υ*_*v*(*i*)**↑**(↓)_ opposes with the sign of υ_*v*(*ii*)**↑**(↓)_, leading to a PC with opposite sign. However, the overall PC *j*_CPGE_(*ω*)_*K*_ and *j*_CPGE_(*ω*)_−*K*_ should be in the same direction as shown in upper panel of Fig. 1, because (*υ*_*c*_*τ*(*ɛ*_*c*_))>((δ*ɛ*_*v*_*/*δ*k*)—*υ*_*v***↑**(↓)_*τ*(*ɛ*_*v*_)). Hence on *σ*+ (*σ*−) excitations on-resonance with these excitons, the PC *j*_CPGE_(*ω*)_σ+ (σ−)_ will be determined by the contributions due to *υ*_*v*(*i*)**↑**(↓)_ and υ_*v*(*ii*)**↑**(↓)_. This will make the main difference between *j*_CPGE_(*ω*)_*K*_ and *j*_CPGE_(*ω*)_−*K*_. Therefore, spin-coupled valley-dependent currents are expected due to excitons formed after light helicity-selective transitions of electrons from the VB to the CB. For off-resonance excitations, the PC expression due to the CPGE is more complicated; *j*_CPGE_(*ω*)_σ+(*σ*−)_ can be *j*_CPGE_(*ω*)_*K*_ or −*K*(−*K* or *K*)=(8*eπ*/*ħ*) [∑_*v*→*c*_ (*υ*_*c*_*τ*(*ɛ*_*c*_))—((δ*ɛ*_*v*_/δ*k*)—*υ*_*v*(**↑**or↓)_*τ*(*ɛ*_*v*_))] |*M*_*v*→*c*_| |*f*(*ɛ*_*c*_)–*f*(*ɛ*_*v*_)| *δ* (*ɛ*_*c*_*–ɛ*_*v*_–*ħω*), in which *v* can be *v*(*i*) (upper VB) or *v*(*ii*) (lower VB). However, we can simply expect that for off-resonance excitation, *j*_CPGE_(*ω*)_σ+_ and *j*_CPGE_(*ω*)_σ−_ will yield similar current values, since the off-resonance excitation simultaneously populates both *K* and −*K* valleys^11^. In the off-resonance excitation, we cannot take advantage of the valley selection rules and spin–valley coupling, which is reflected in the variance between *j*_CPGE_(*ω*)_σ+_ and *j*_CPGE_(*ω*)_σ−_ in on-resonance excitations. This underlines the importance of strong excitonic character that can be obtained in high-quality samples to observe a pronounced difference between *j*_CPGE_(*ω*)_σ+_ and *j*_CPGE_(*ω*)_σ−_.

### Device characterization and photocurrent

**Figure 2:**
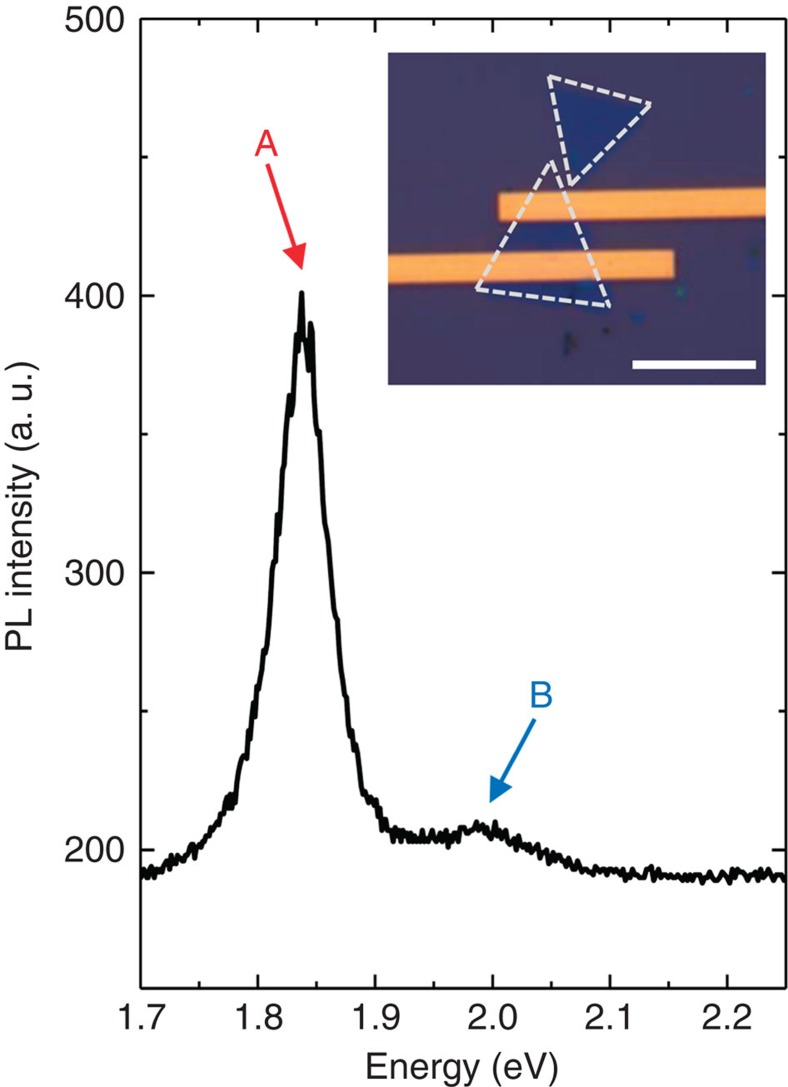
Photoluminescence of the sample. The strong excitonic *A* peak is at 1.84 eV. The other excitonic *B* peak is at 2 eV. The respective optical image is shown in the inset. The dash lines in the inset are drawn to make easier to visualize the flakes. The orange bars are Ti/Au contacts. Scale bar, 5 μm.

**Figure 3:**
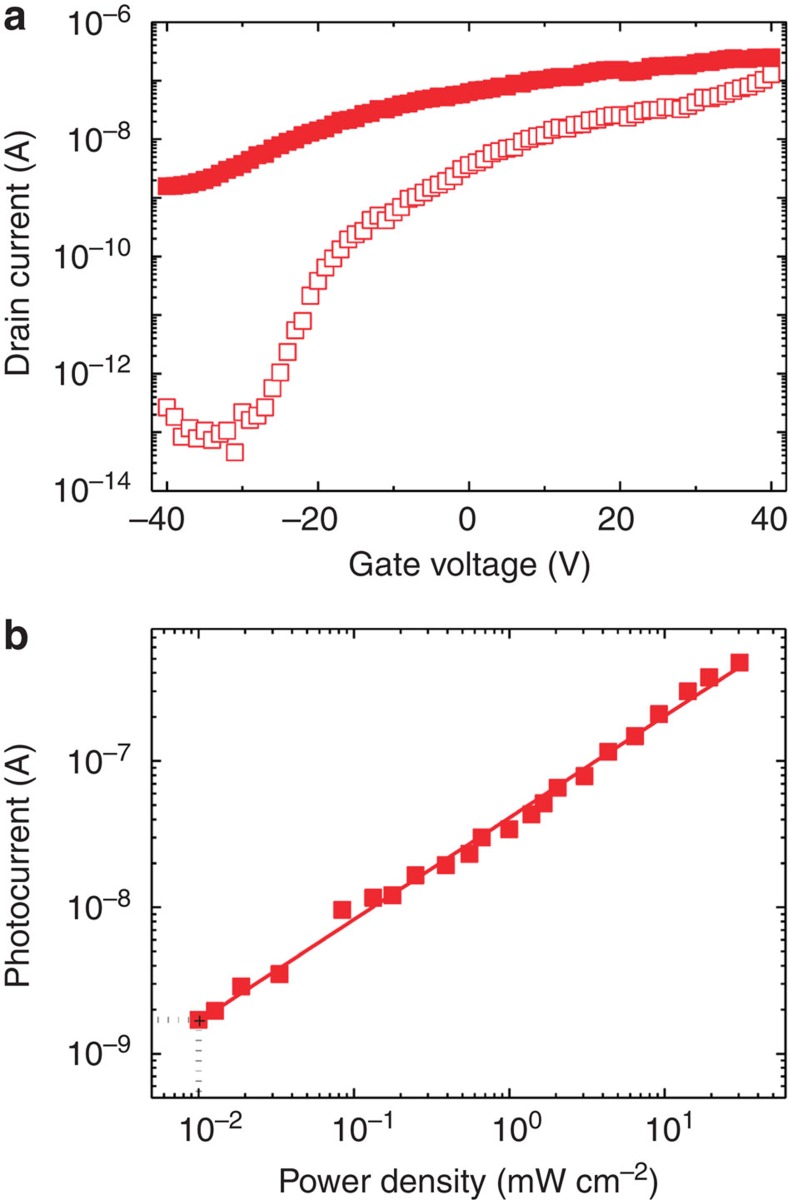
Device characterization of the sample. (**a**) Gate voltage dependence of drain current, at 10 V drain voltage, in dark (empty squares) and upon 20 mW cm^−2^ illumination by 2.33 eV laser (full squares). The ratio of *I*_Illumination_ to *I*_Dark_ is about 10^4^ in the off state (*V*_g_=−35 V) (**b**) Photocurrent measured as a function of laser power density at *V*_g_=−40 V is shown. The data (full squares) are fit to a straight line and a photoresponsivity (*R*) of 3.5 A W^−1^ is obtained.

Figure 2 shows the microphotoluminescence of the device prepared by CVD as shown in the inset. The microphotoluminescence measurement was performed by 532-nm laser excitation at room temperature. The strong excitonic peak of *A* (*B*) exciton corresponds to pair of an electron in the CB and a hole in the upper (lower) band of the split VB at *K* valleys. The PL peak for *A* (*B*) excitons in these samples is around 1.84 eV (∼2 eV) and the *A* excitons are much stronger in intensity. The quality of the flakes was demonstrated by Raman microspectroscopy and contrast-enhanced fluorescence optical microscope image in Supplementary Figs 1 and 2, respectively. Initial electrical measurements of monolayer MoS_2_ field-effect transistors were performed in dark. Drain current versus drain voltage (*V*_d_) characteristics, at zero gate voltage (*V*_g_), are shown in Supplementary Fig. 3. For 10 V applied *V*_d_, the field-effect transistors characteristics mark an on-state at −30 V (Fig. 3a). By a linear fit between 30 and 40 V, we can estimate the mobility of these devices from *μ*=(*L*/*W*) (d*I*/d*V*) (1/*CV*_d_). Here *L* is the device length, *W* the device width and *C* is the back gate capacitance. The mobility is ∼0.5 cm^2^ V^−1^ s^−1^, as shown in Supplementary Fig. 4, which is in agreement with the reported values^18^. Subsequent drain current measurements on illumination by 2.33 eV laser with 20 mW cm^−2^ power density, and comparison with the dark measurements, are illustrated as a function of *V*_d_ in Supplementary Fig. 5. The drain current as a function of *V*_g_, on illumination (*I*_Illumination_) and in dark (*I*_Dark_) are compared in Fig. 3a. The ratio of *I*_Illumination_/*I*_Dark_ is ∼10^4^ in the off state (*V*_g_=−35 V).

Laser power density dependence of drain current at −40 V *V*_g_ and 10 V *V*_d_ was measured. Figure 3 shows PC (*I*_Illumination_—*I*_Dark_) versus laser power density. Photoresponsivity (*R*) at the minimum power for the device shown in the inset to Fig. 2 is about 3.5 A W^−1^, which is about two orders of magnitude lower than the reported one^18^. The power dependence of the measured drain current (*I*_d_) can be fitted according to *I*_d_ ∝ (Laser Power)^*α*^, where *α* is about 0.7 for our samples and the reported MoS_2_^18^. At higher power densities, *R* drops either due to the trap states in MoS_2_ or the interface of MoS_2_ and the substrate^27^. Therefore, to elucidate the photoinduced transport effects of monolayer MoS_2_, further PC measurements at both high and low laser power densities were performed using conventional amplitude modulation and lock-in detection.

**Figure 4:**
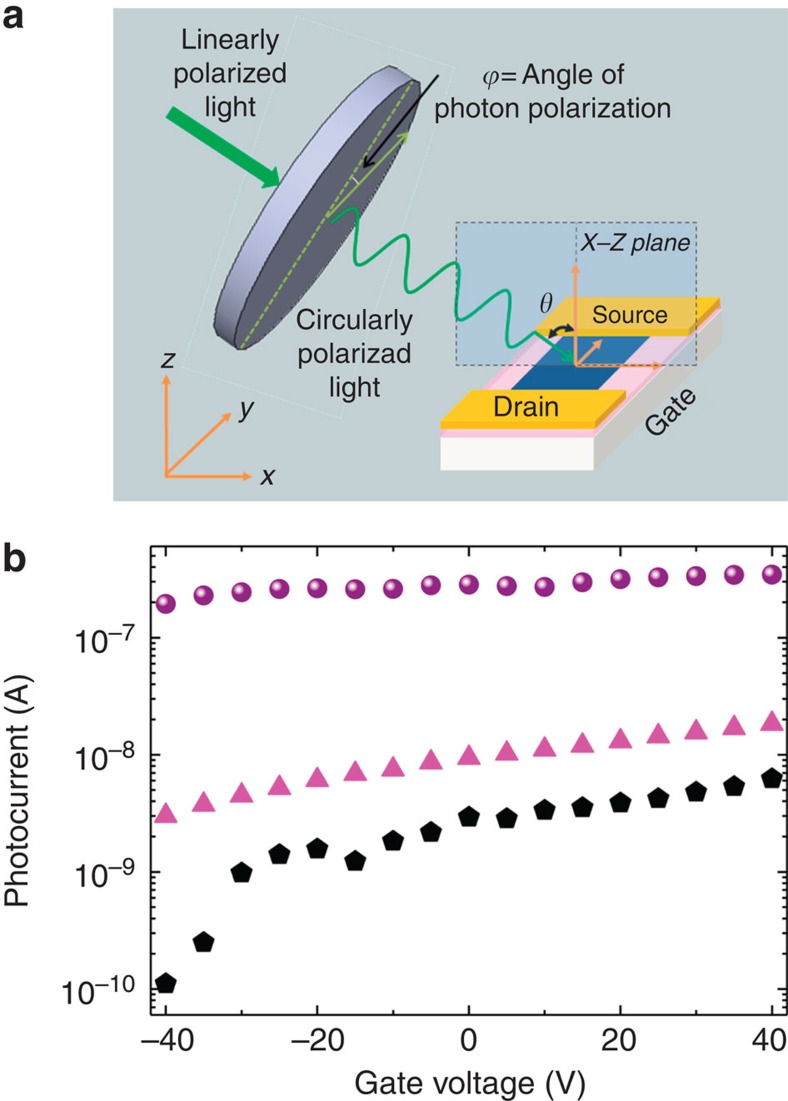
Experimental configuration and photocurrent. (**a**) Description of photocurrent setup using the lock-in technique to collect the photocurrent (PC) amplitude and phase is given. Using a linearly polarized laser with a quarter-wave plate (QWP), circularly polarized light obtained and directed on the sample with an oblique angle *θ*. By rotating QWP it is possible to change the angle of photon polarization *ϕ*. (**b**) PC of the sample illuminated by 2.33 eV laser, for *ϕ*=0° and *θ*=45°. Spheres are the data for 10 V drain voltage and 9.78 W cm^−2^ laser power density. Up triangles are the data for 10 V drain voltage and 60 mW cm^−2^. Pentagons are the data for 0.7 V drain voltage and 60 mW cm^−2^. Helicity-dependent PC was performed in this regime (off state) in which there are only photogalvanic and photon drag effects.

In Fig. 4b, gate dependence of PC signal for different *V*_d_ and laser power density with 2.33-eV excitation is presented for *ϕ*=0° and *θ*=45° configuration as described in the experimental setup of Fig. 4a. Both PC signal amplitude and phase were measured. Phase is around 0° while the *V*_g_, *V*_d_ and laser power were varied; no sign change of PC was observed as shown in Supplementary Fig. 6. At high *V*_d_ of 10 V, both high-power-density data (9.78 W cm^−2^) and low-power-density data (60 mW cm^−2^) are being affected by bolometric effects as in the AB-stacked bilayer graphene^28^, which are due to the high channel doping. The PC amplitude increases almost linearly from 100 to 200 nA from −40 V to 40 V for high power density; from 3 to 20 nA for low power density. At low *V*_d_ of 0.7 V and low power density (60 mW cm^−2^), the measured PC is dominated by photogalvanic effect, photon drag effect and low channel doping. The PC amplitude increases logarithmically from 0.1 to 8 nA, in accordance with the gate dependence of drain current data in Fig. 4b. Low *V*_d_ also prevents from artificial PC such as photogating effects^29^ and photothermal effects^30^. Therefore, intrinsic photogalvanic effects are expected to appear at low *V*_d_ and low illumination intensity. Here we focus on this regime to address subtle effects of light helicity on spin–valley PCs, which has been a subject of fundamental opto-spintronic applications^31^,^32^,^33^.

### Helicity-dependent PC by 2.33 and 1.96 eV excitations

**Figure 5:**
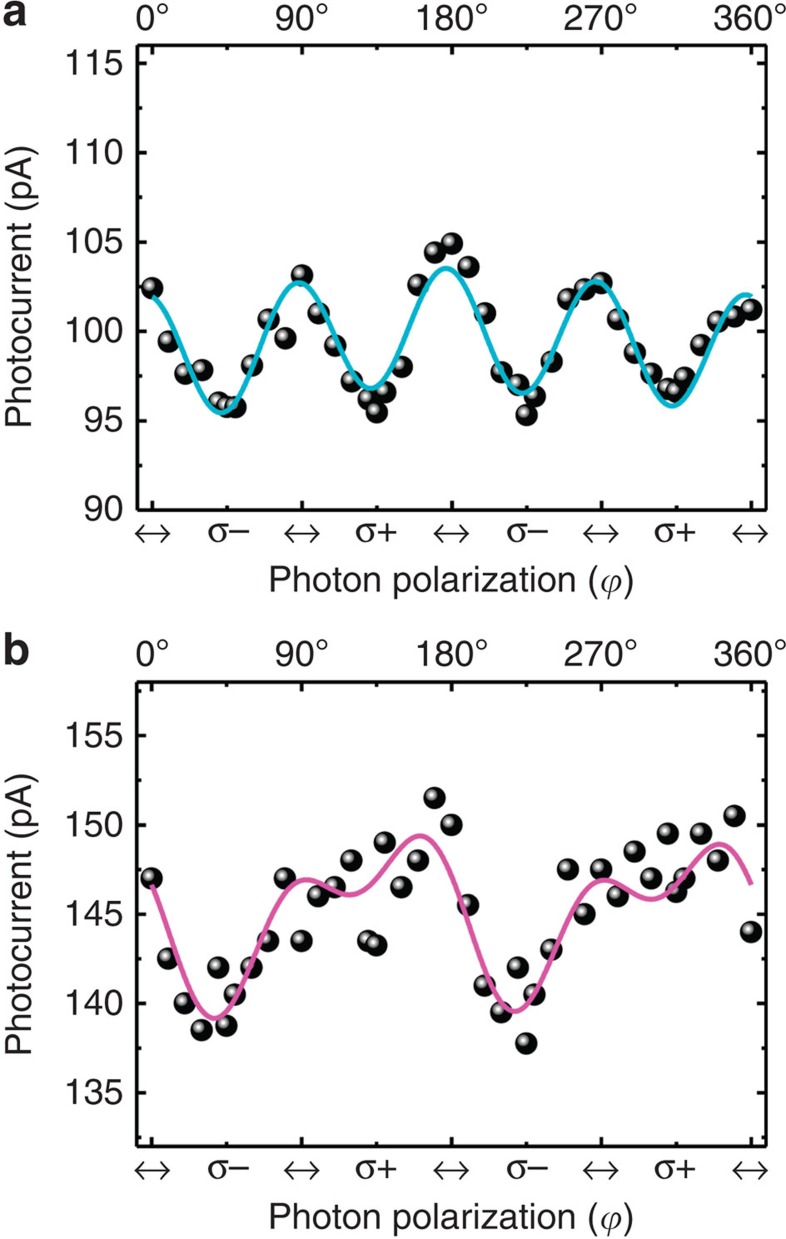
Spin-coupled valley-dependent dichroic photocurrent in monolayer MoS_2_. (**a**) Photocurrent (PC) as a function of angle of photon polarization *ϕ* when illuminated by 2.33 eV (off-resonance with the excitons) laser is shown. The PC data are collected by rotating a quarter-wave plate which changes polarization of a linearly polarized laser from 0° linear (↔) to 45° left circular (*σ*−), back to 90° linear (↔) to 135° right circular (*σ*+), and then back to 180° linear (↔) polarization, and so on. The (cyan) curve is the fitting function based on the phenomenological PC formula for monolayer MoS_2_, yielding negligible polarization between *σ*+ and *σ*− excitations. (**b**) PC as a function of *ϕ* when illuminated by 1.96 eV (on-resonance) laser. The (magenta) curve is the fitting function based on the phenomenological PC formula for monolayer MoS_2_, yielding a polarization of ∼60% between *σ*+ and *σ*− excitations, which control the valley currents in *K* (linked with hole spin up) and −*K* (linked with hole spin down) valleys, respectively.

Figure 5 shows our light helicity-dependent PC results by varying the angle of photon polarization (*ϕ*) for off-resonance (2.33 eV photon energy, 0.7 V drain voltage and 60 mW cm^−2^ illumination intensity) and for on-resonance excitation (1.96 eV photon energy, 2 V drain voltage and 100 mW cm^−2^ illumination intensity) at *V*_g_=−40 V. As expected, for off-resonance excitation, (Fig. 5a) the PC exhibits no clear dependence on angle of photon polarization (that is, *σ*+ and *σ*− excitation). Indeed, there is no preferential optical transition (due to electron–photon interaction and scattering events) from the upper or lower VBs to the CB, in terms of valleys, on absorption of photon energies off-resonance with the excitonic transition. The data are fitted to the phenomenological formula *J*=*C*_1_ sin 2*ϕ*+*L*_1_ sin 4*ϕ*+*L*_2_ cos 4*ϕ*+*D* and the fitting parameters are tabulated in Table 1. The component of PC response related to circularly polarized light is the *j*_CPGE_. But for this excitation, *j*_LPDE_ is the dominant contribution. Thus, by subtracting *j*_LPDE_ and polarization-independent term from the measured PC, PC (*σ*±), one can obtain *j*_CPGE_ (*σ*±)=PC (*σ*±)—(*−L*_2_+*D*). Then PC polarization can be defined as *P*=[|*j*_CPGE_ (*σ*+)|−|*j*_CPGE_ (*σ*−)|]/[|*j*_CPGE_ (*σ*+)|+|*j*_CPGE_ (*σ*−)|]. By using the parameters in Table 1, *P* is 0.8±0.4 %. On the basis of microscopic description of the CPGE valley current, *υ*_*v*(*i*)_- and *υ*_*v*(*ii*)_-dependent terms cancel each other and eventually results in a negligible PC polarization.

The picture changes markedly in the case of PC observed for excitation with photon energy=1.96 eV, which is on-resonance with the excitonic transitions. This photon energy is on-resonance mainly with *A* excitons, as explained in Supplementary Note 1. The angle of photon polarization dependence of PC exhibits a clear difference between *σ*+ and *σ*− excitations in Fig. 5b, as expected for spin-coupled valley currents with resonant excitation. As shown in Table 1, the parameter *C*_1_ is much larger compared with the 2.33-eV excitation, although *L*_1_ still small and *L*_2_ gets relatively smaller. The coefficients *C*_1_ and *L*_2_ stand as the most prominent ones in determining polarization-dependent PC(*σ*±). The parameter *C*_1_ becomes negligible for the on-resonance excitation once the angle of incidence *θ*=0°, as shown in Supplementary Fig. 7 and Supplementary Table 1. The figure of merit for PC polarization can be taken as the ratio of |*C*_1_|/|*L*_2_|, which is 0.06 for off-resonance (2.33 eV) excitation and 1.94 for on-resonance (1.96 eV) excitation. In terms of microscopic origin, the PC polarization for the excitation on resonance with the *A* excitons can be qualitatively given as ∼|*υ*_*v*(*i*)_*τ*(*ɛ*_*v*_)|/|*υ*_*c*_*τ*(*ɛ*_*c*_)| based on the *j*_CPGE_ defined earlier in Fig. 1. In other words, *j*_CPGE_ dominates and the concomitant *P* has high values of 60±30% when excited by 1.96 eV. This value is larger when compared with PL polarization at zero *V*_g_ presented in Supplementary Fig. 8. This could be due to loss of polarization during the process of circularly polarized excitation and collection in PL measurements. Also, in PC measurements we are able to identify purely the CPGE-dependent valley current and additional effects in PL are suppressed. The large error in *P* is due to the fluctuations in the data as explained in Supplementary Note 2. It is possible to obtain a better signal by further improvement of device fabrication. As a control experiment, we used the same experimental setup at *θ*=45° with on-resonance laser, only by replacing circularly polarized light by linearly polarized light (we used half-wave plate instead of QWP); the data at 45°, 135°, 225° and 315° yield similar values, unlike circularly polarized excitation. We also give a comparison of our results of the CVD grown monolayer MoS_2_ sample with the results of a mechanically cleaved monolayer MoS_2_ sample in Supplementary Figs 9 and 10, by electrical measurements in Supplementary Figs 3, 4 and 11, photoresponse in Supplementary Fig. 12, circularly polarized PL in Supplementary Fig. 13 and light helicity-dependent spin–valley current in Supplementary Fig. 14 and in Supplementary Table 2. In mechanically cleaved sample, the PC polarization that reflects the spin–valley coupling is relatively smaller than the CVD sample as explained in Supplementary Note 2, which makes CVD grown MoS_2_ devices promising for spin-dependent opto-valleytronic applications.

## Discussion

Our results of exciton-related spin-coupled valley-dependent PC are consistent with helicity-dependent PL measurements^11^ and recent observation of the valley Hall effect in monolayer MoS_2_^34^. These results may have a number of applications in nanoeletronics and photonics, such as such as polarization-sensitive light-detection schemes or integrated spintronic devices. Furthermore, more examples can be developed based on magnetoelectric effects of bilayer TMDs^35^ and spin–orbit interaction in graphene/TMD hybrid systems^36^, since CVD grown TMD devices are promising in terms of electrical transport^37^. Thanks to room-temperature operation, the control over spin-dependent valley PC is more application oriented than the recently discovered helicity-dependent PC of topological insulators^38^. Moreover, special attention should be paid to biological environments, which are sensitive to light handedness and colour. Examples can be given in the area of light-harvesting chlorophyll (LHC) research^39^ and biomolecule–nanoparticle interaction^40^. For instance, light helicitiy dependence has been demonstrated for the chiral LHC, which functions around red colour (633 nm—1.96 eV) wavelength but insensitive to green colour^41^. This realizes the possibility of integrating chiral LHC with ultrasensitive spin–valley-coupled MoS_2_ phototransistors. Also, the biophysical community has been investigating the protein binding to nanomaterials for vision-related nanomedical purposes^42^. On the basis of our observations, we believe it is viable to utilize the unique optoelectronic features of MoS_2_ in the exploration of light sensitive bio–nano systems.

We emphasize that high-quality monolayer MoS_2_, which is a spin–valley coupling system grown by chemical vapour deposition fabricated as phototransistor exhibits helicity-dependent PC behaviour, based on its unique symmetry conditions. Spin-dependent valley currents can be generated by circularly polarized laser, and can be manipulated by laser excitation and electrical gating. We were able to demonstrate that in *K* or −*K* valleys with given spin (up or down, respectively) exciton-related electron conduction can take place, and PC polarization as high as 60% was obtained by choosing a suitable visible laser with a known circular polarization (*σ*+ or *σ*−). The observed PC polarization degree is even higher than the PL polarization since we are able to resolve purely the CPGE-dependent valley current. The findings in this work-shed light into the intrinsic photogalvanic effects and helicity-selective transitions in monolayer transition metal dichalcogenides. Besides their fundamental importance, we believe these results may pave the way for application of opto-valleytronic device concepts in interdisciplinary research spanning from nanoelectronics and photonics to biotechnologies.

## Methods

### Sample growth and device processing

Monolayer MoS_2_ flake with size ∼5 μm is grown by a CVD method (as explained in detail elsewhere^24^) onto Si substrate with 300-nm SiO_2_. The triangular flake was picked among homogeneous, high crystalline quality flakes then processed into a device, by e-beam lithography with Ti (5 nm)/Au (75 nm) contacts for electrical and PC measurements.

### PC measurements

By using lock-in technique with a low noise current preamplifier, PC amplitude and phase have been measured for monolayer MoS_2_ phototransistors at room temperature. The linearly polarized laser beam is centred on the sample with an oblique angle *θ* after being chopped to a frequency of 137 Hz, which is the frequency of the measured current at the same time. As shown in Fig. 4, the PC setup has the option to circularly polarize the linearly polarized light field by using a QWP. The angle of photon polarization, *ϕ*, can be varied by rotating the QWP.

## Additional information

**How to cite this article:** Eginligil, M. *et al.* Dichroic spin–valley photocurrent in monolayer molybdenum disulphide. *Nat. Commun.* 6:7636 doi: 10.1038/ncomms8636 (2015).

## Supplementary Material

Supplementary Information Supplementary Figures 1-14, Supplementary Tables 1-2, Supplementary Notes 1-2 and Supplementary References.

## Figures and Tables

**Table 1:** Photocurrent components of the sample excited by circularly polarized light.

	***C***_**1**_	***L***_**1**_	***L***_**2**_	***D***
1.96 eV	−3.5	−1.5	1.8	145.1
2.33 eV	−0.2	−0.7	3.2	99.5

Parameters are determined from the phenomenological photocurrent (PC) formula fittings for the monolayer MoS_2_ grown by chemical vapor deposition. The PC components are obtained by 1.96 and 2.33 eV circularly polarized excitations.
