# Supplementary material for: Dichroic spin–valley photocurrent in monolayer molybdenum disulphide
Source: Nat Commun. 2015 Jul 2;6:7636. doi: 10.1038/ncomms8636 (PMC4506497; doi:10.1038/ncomms8636)
Supplement: Supplementary Information — Supplementary Figures 1-14, Supplementary Tables 1-2, Supplementary Notes 1-2 and Supplementary References. [file ncomms8636-s1.pdf]

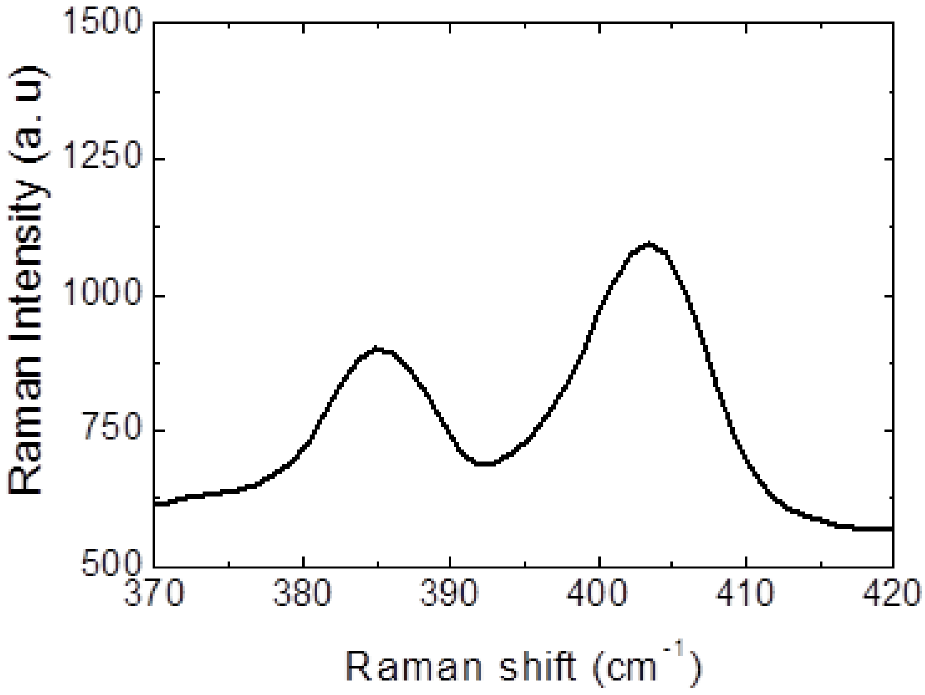

Supplementary Figure 1 | Raman spectrum of monolayer MoS_2 grown by chemical vapour deposition. Raman peaks of the monolayer sample grown by chemical vapour deposition (S-CVD) are E^1_{2g} peak which is at 385 cm^{-1} and A_{1g} peak which is at 403 cm^{-1}. These values are consistent with the published work[1].

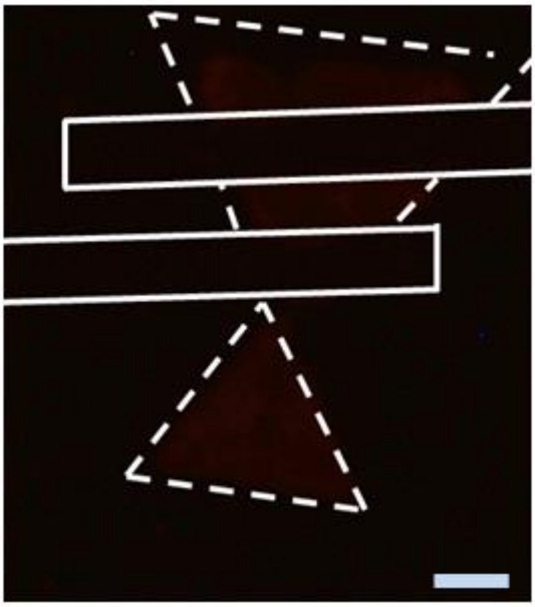

Supplementary Figure 2 | Fluorescence image of monolayer MoS_2 grown by chemical vapour deposition. Solid lines show the Ti/Au contacts. Dash (white) lines show the flake. The scale at the bottom right is 1 μm. The fluorescence image of the device grown by chemical vapour deposition is clear.

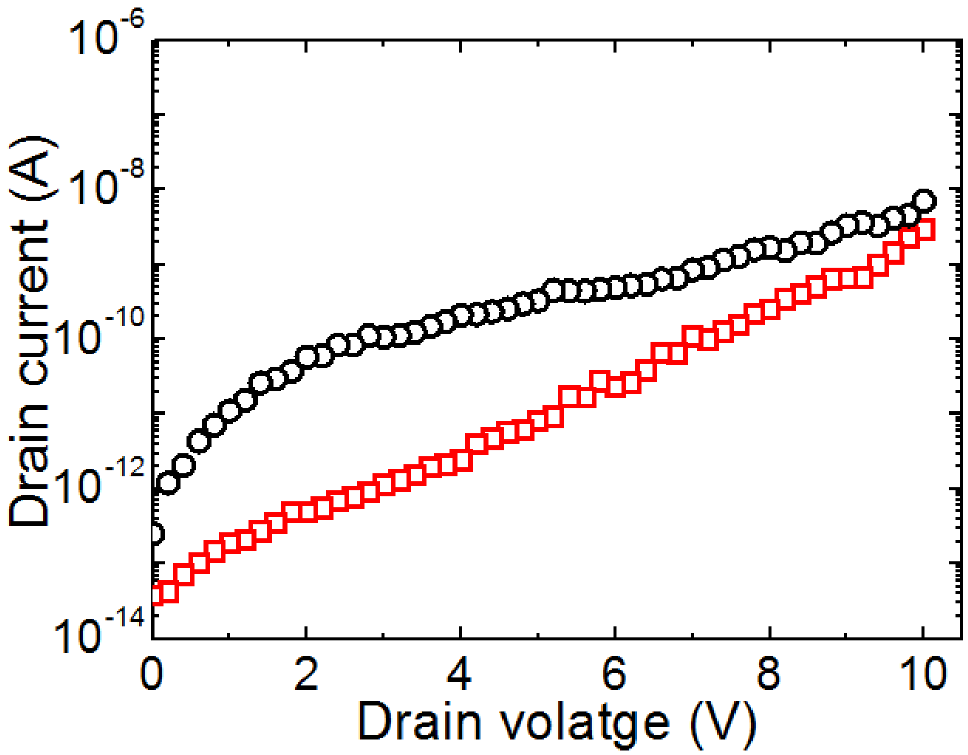

Supplementary Figure 3 | Drain current vs. drain voltage characteristics of monolayer MoS_2 field-effect transistors at zero gate voltage at dark. Drain current vs. drain voltage characteristics of monolayer MoS_2 field-effect transistors. Black empty circles are the data for the sample grown by chemical vapour deposition (S-CVD). Red empty squares are the data for the sample prepared by mechanical cleaving (S-MC). Both S-CVD and S-MC data are taken at zero gate voltage at dark, and shown in logarithmic scale.

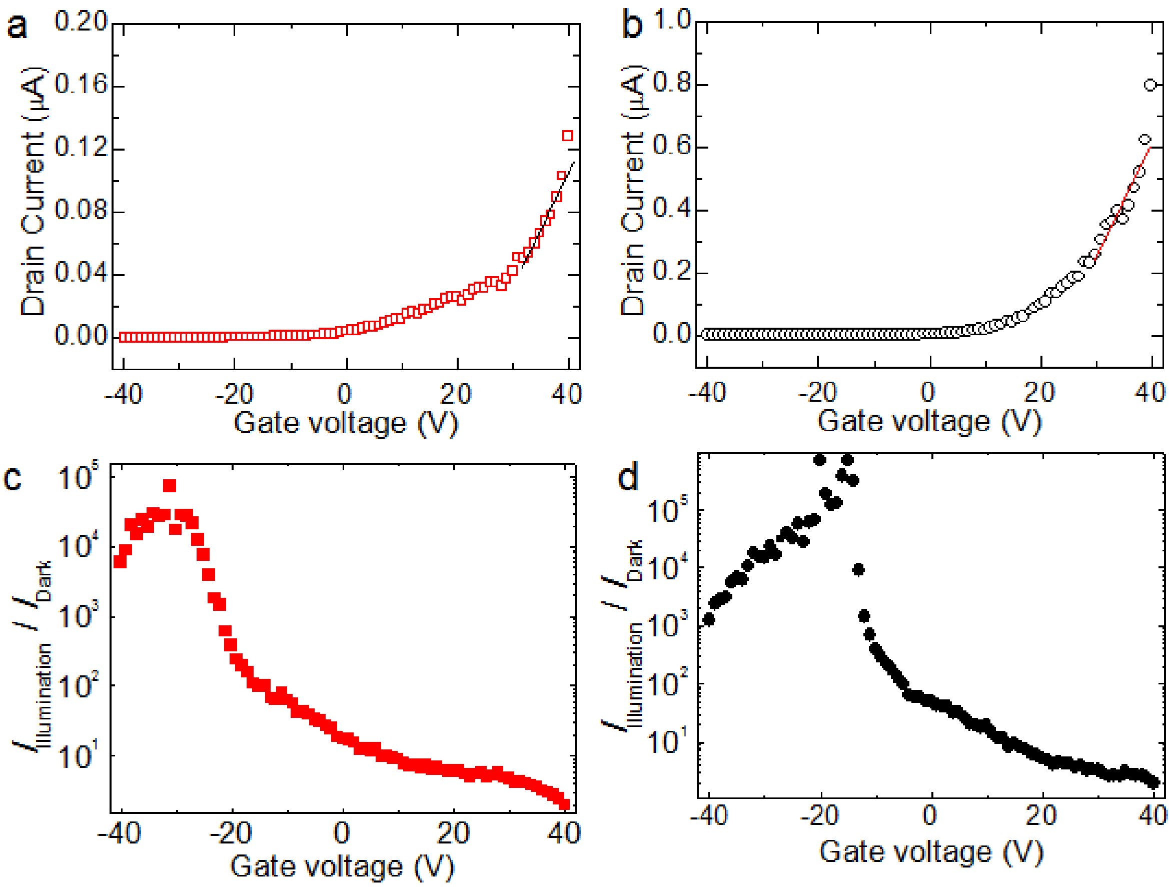

Supplementary Figure 4 | Mobility of monolayer MoS_2 samples and drain current ratios for light ON and OFF. a Gate voltage dependence of drain current for the sample grown by chemical vapor deposition (S-CVD) and **b** for the sample prepared by mechanical cleaving (S-MC), at dark and 10 V drain voltage. Mobility of the samples is determined by fitting a straight line to the data between 30 V – 40 V. The mobilities of S-CVD and S-MC are approximately 0.5 and 9 $cm^2 V^{-1} s^{-1}$, which are in agreement with the reported values[3]. **c** Gate voltage dependence of the ratio of drain current obtained upon illumination (illuminated by 2.33 eV laser with a power density of 20 mW cm^{-2}) to that obtained in dark for S-CVD and **d** for S-MC are given. Both devices exhibit a ratio of $I_{Illumination}$ / I_{Dark} ~ 10^4 in the off state, while S-MC exhibits large variations in the off state. This may be considered as a consequence of localization and less effective screening of charge carriers[4] or midgap states[5] in S-MC.

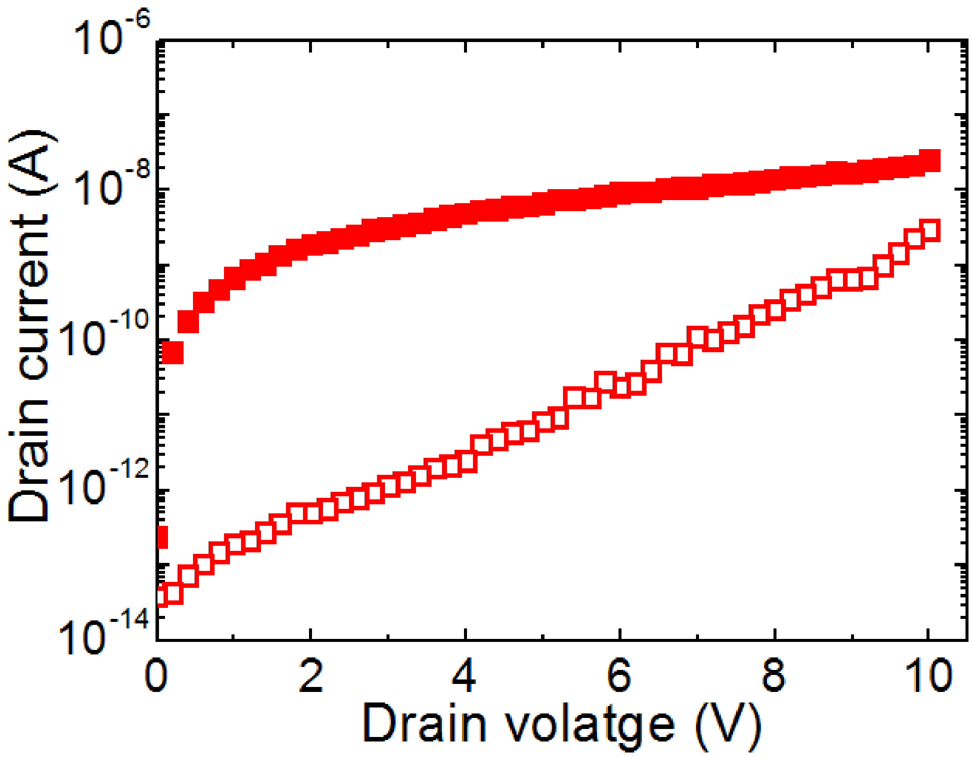

Supplementary Figure 5 | Drain current vs. drain voltage characteristics of monolayer MoS_2 sample grown by chemical vapour deposition at dark and illumination. Drain current vs. drain voltage characteristics of monolayer MoS_2 field-effect transistor grown by chemical vapour deposition, S-CVD, at zero gate voltage, in logarithmic scale. Empty squares show the data at dark, full squares show the data at 20 mW cm^{-2} illumination by 2.33 eV laser excitation.

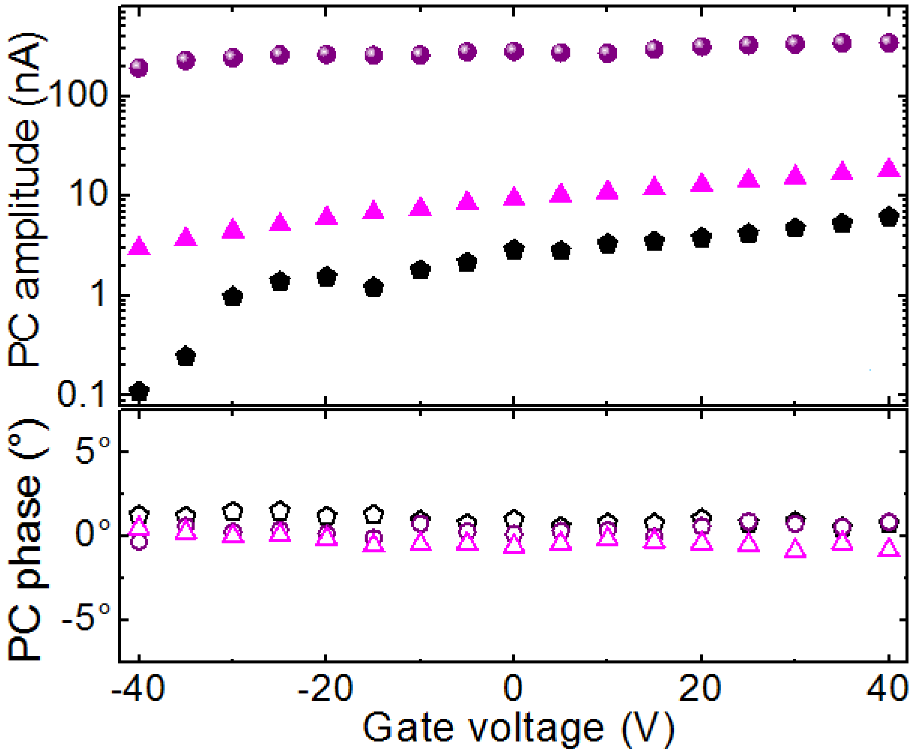

Supplementary Figure 6 | Photocurrent measurements of monolayer MoS_2 grown by chemical vapour deposition. a Photocurrent amplitude and **b** phase for the sample grown by chemical vapor deposition, S-CVD, illuminated by 2.33 eV laser, for $\varphi = 0^{\circ}$ and $\theta = 45^{\circ}$are given. In **a,** spheres are the data for 10 V drain voltage and 9.78 W cm^{-2} laser power density. Up triangles are the data for 10 V drain voltage and 60 mW cm^{-2}. Pentagons are the data for 0.7 V drain voltage and 60 mW cm^{-2}. All the phase data in **b** (open spheres, open up triangles, and pentagons data correspond to the data in **a**) are close to be 0˚.

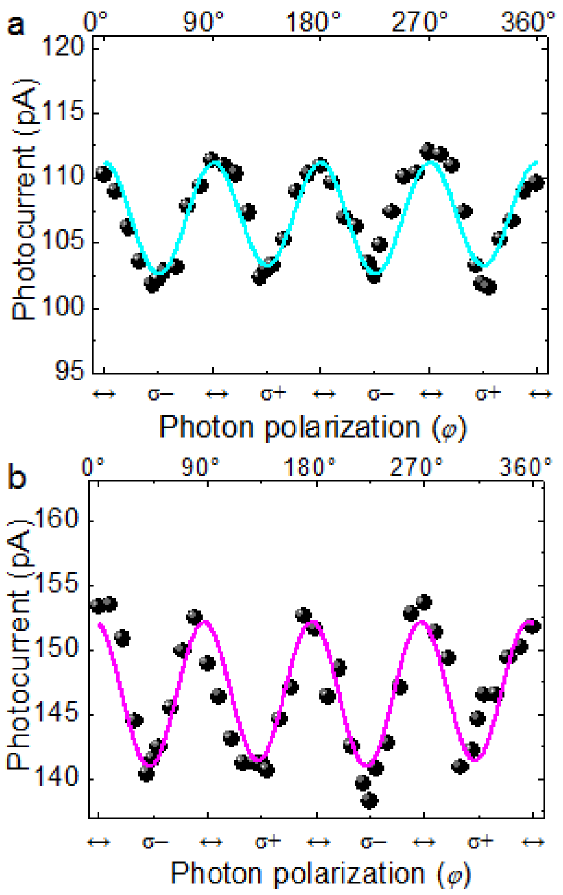

Supplementary Figure 7 | Light helicity dependent photocurrent in monolayer MoS_2 grown by chemical vapor deposition for normal incidence. a Photocurrent (PC) as a function of angle of photon polarization φ is shown for the monolayer MoS_2 grown by chemical vapor deposition (S-CVD) when illuminated by 2.33 eV (off-resonance with excitons) laser at $\theta = 0^{\circ}$. The (green) curve is the fitting function based on the phenomenological PC formula for monolayer MoS_2, yielding negligible PC polarization. **b** PC as a function of angle of incidence of polarization φ is shown for S-CVD when illuminated by 1.96 eV (on-resonance with ***A*** exciton) laser. The (red) curve is the fitting function based on the phenomenological PC formula for monolayer MoS_2, yielding a similarly negligible PC polarization. These data provide evidence for spin-valley PC due to the differences of the CPGE observed for σ+ and σ– excitation only for $\theta \neq 0^{\circ}$, and for laser on-resonance with ***A*** exciton.

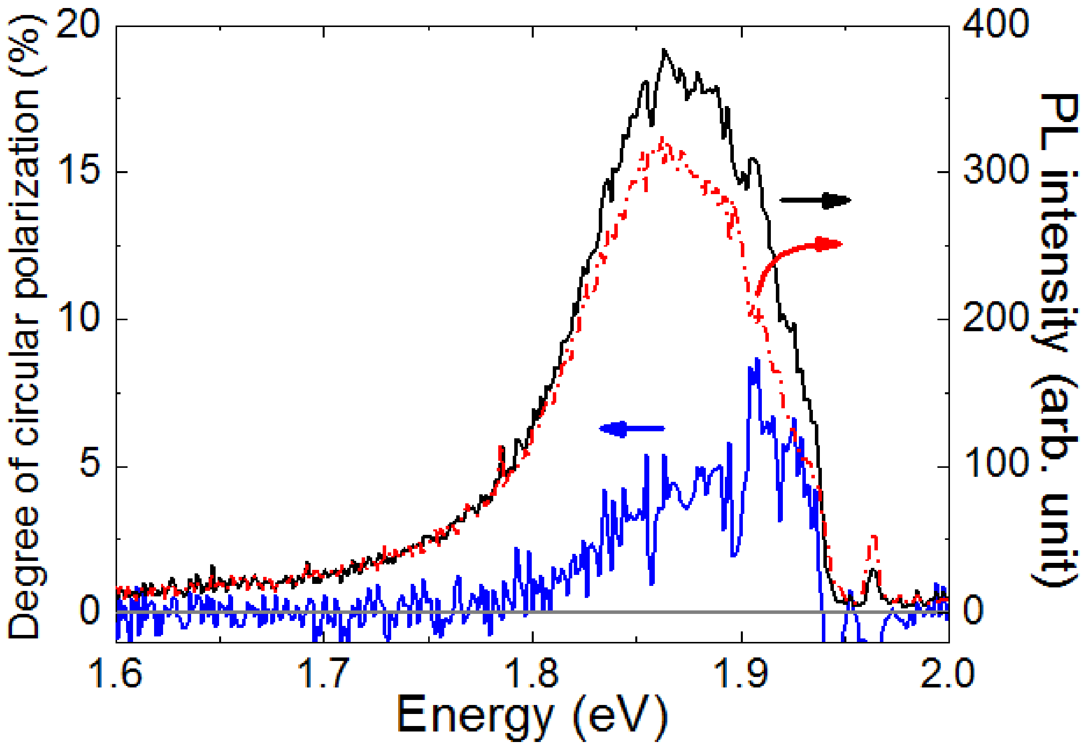

Supplementary Figure 8 | Circularly polarized photoluminescence of monolayer MoS_2 grown by chemical vapour deposition. The peak photoluminescence (PL) of the sample grown by chemical vapour deposition (S-CVD) is at 1.865 eV. The PL intensity of S-CVD for both left (black solid curve) and right (red dash dot curve) circularly polarized PL upon excitation with left circularly polarized light is shown on the right axis. On the left axis degree of circular polarization (blue solid curve) yields a PL polarization of ~ 10 %.

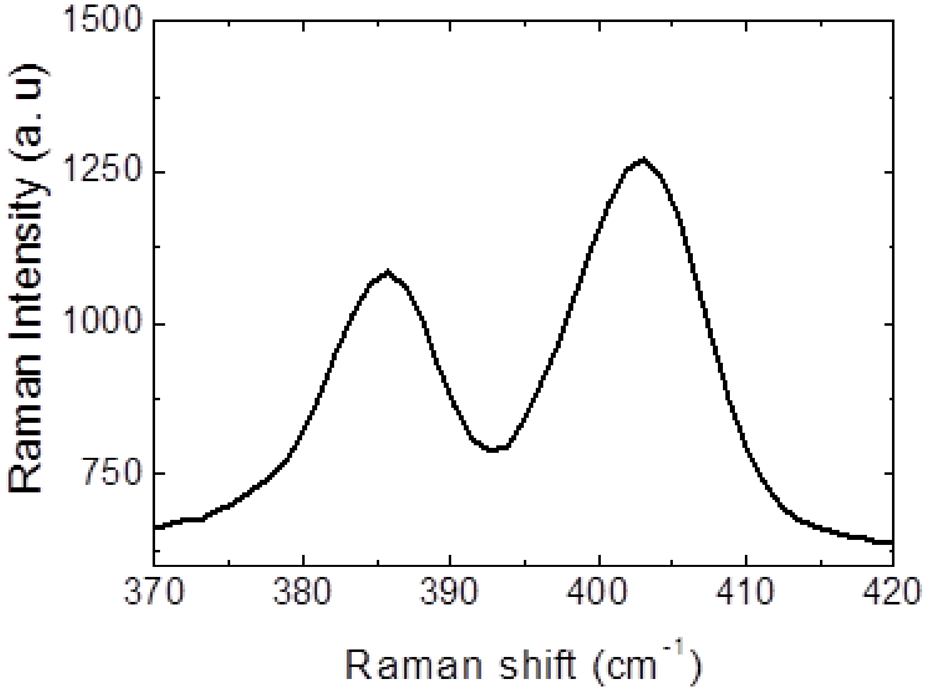

Supplementary Figure 9 | Raman spectrum of mechanically cleaved monolayer MoS_2. Raman peaks of the mechanically cleaved sample (S-MC) are E_{2g}^{1} peak which is at 385 cm^{-1} and A_{1g} peak which is at 403 cm^{-1}. The spectrum is similar to that of the sample grown by chemical vapour deposition, S-CVD.

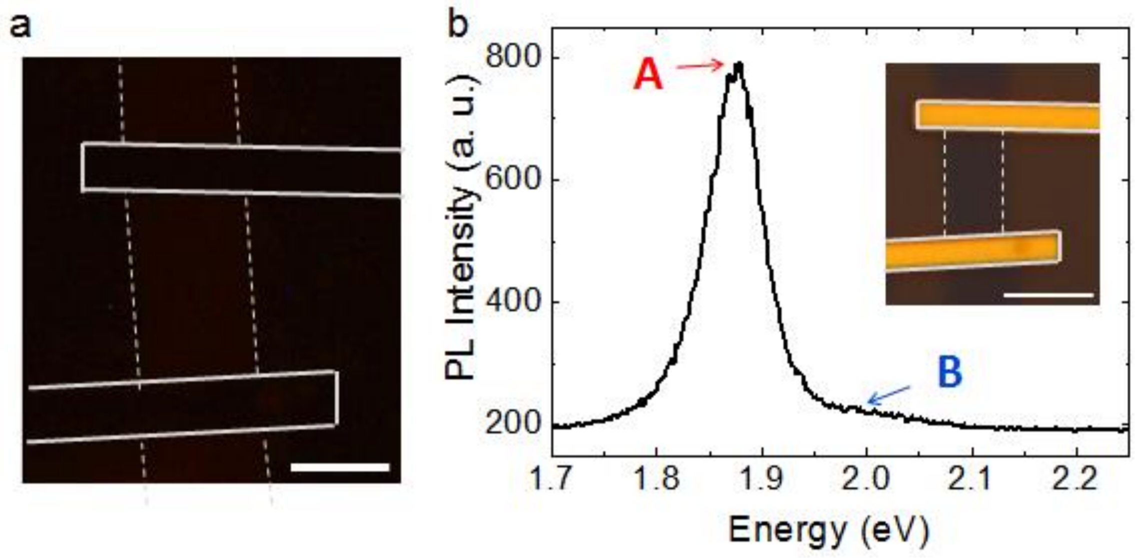

Supplementary Figure 10 | Fluorescence image, optical image and photoluminescence of monolayer MoS_2 prepared by mechanical cleaving. a Fluorescence image of the sample prepared by mechanical cleaving (S-MC). Solid lines show the Ti/Au contacts. Dash lines show the flake. The scale bar is 1 μm. The fluorescence image is relatively weaker than the sample grown by chemical vapour deposition (S-CVD). **b** Photoluminescence of S-MC is shown. The strong direct excitonic ***A*** peak is at 1.89 eV for S-MC. The peak intensity is doubled in S-MC and a blueshift of ~50 meV observed, compared to S-CVD. This could be due to modulated n-type doping for S-MC sample[2]. The other direct excitonic ***B*** peak is at 1.98 eV for both S-MC and S-CVD. The respective optical image is shown in the inset. The dash lines in the inset are drawn to make easier to visualize the flakes. The orange bars are the Ti/Au contacts. The scale bar is 2 μm.

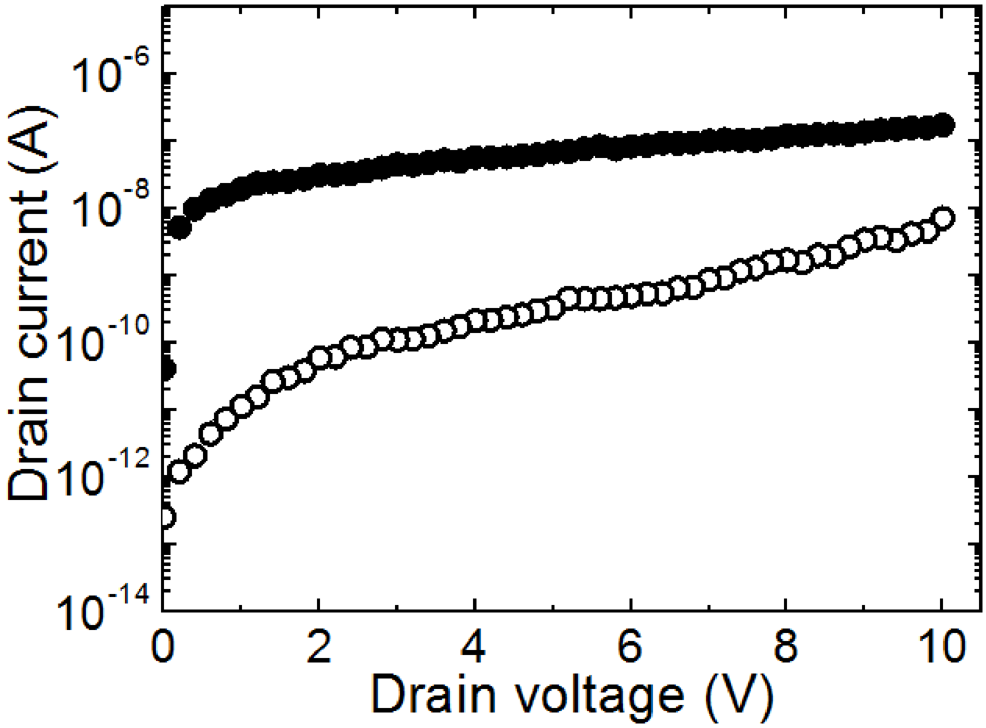

Supplementary Figure 11 | Drain current vs. drain voltage characteristics of monolayer MoS_2 sample prepared by mechanical cleaving at dark and at illumination. Drain current vs. drain voltage characteristics of mechanically cleaved monolayer MoS_2 field-effect transistor S-MC, at zero gate voltage, in logarithmic scale. Empty circles denote the data at dark, full circle show the data at 20 mW cm^{-2} illumination by 2.33 eV laser excitation.

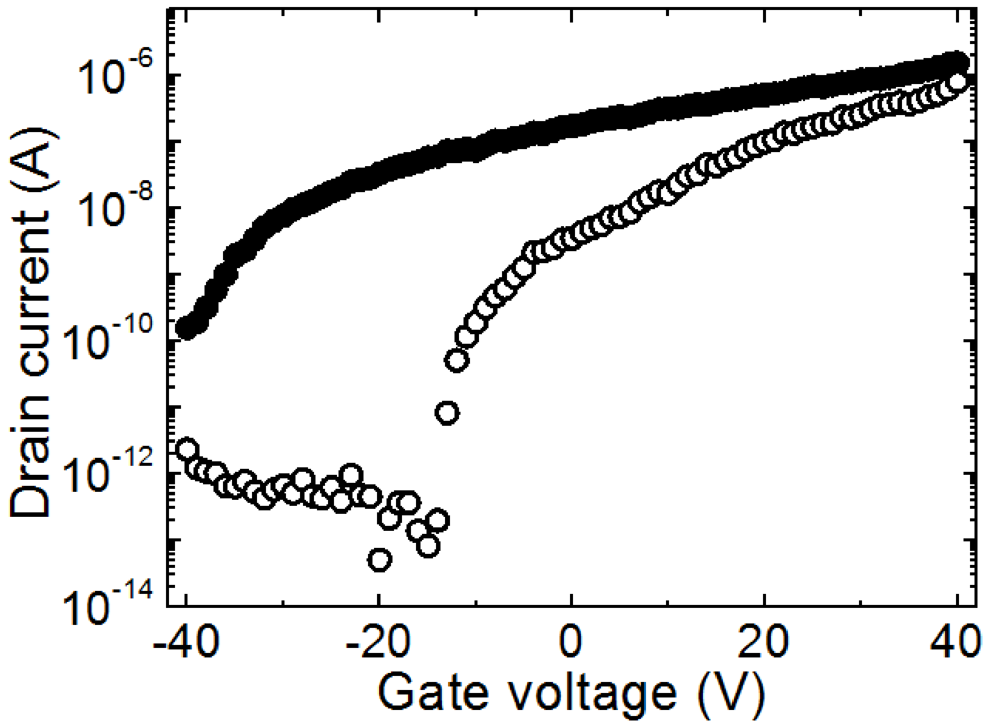

Supplementary Figure 12 | Drain current vs. gate voltage characteristics of monolayer MoS_2 prepared by mechanical cleaving at dark and at illumination. Drain current vs. gate voltage characteristics of mechanically cleaved monolayer MoS_2 field-effect transistor S-MC, at 10 V drain voltage, in logarithmic scale. Empty circles show the data at dark, full circles show the data at 20 mW cm^{-2} illumination by 2.33 eV laser excitation.

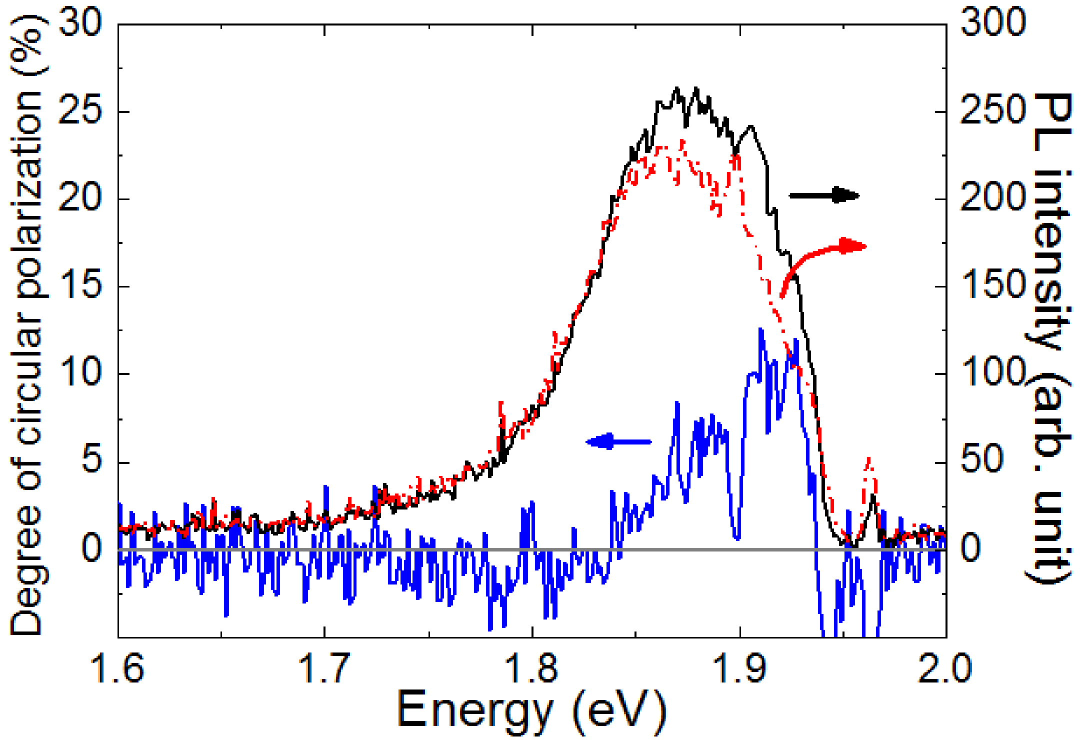

Supplementary Figure 13 | Circularly polarized photoluminescence of monolayer MoS_2 prepared by mechanical cleaving. The peak photoluminescence (PL) of monolayer MoS_2 prepared by mechanical cleaving (S-MC) is at 1.865 eV. The PL intensity of S-MC for both left (black solid curve) and right (red dash dot curve) circularly polarized PL upon excitation with left circularly polarized light is shown on the right axis. On the left axis degree of circular polarization (blue solid curve) yields a PL polarization of ~ 10 %.

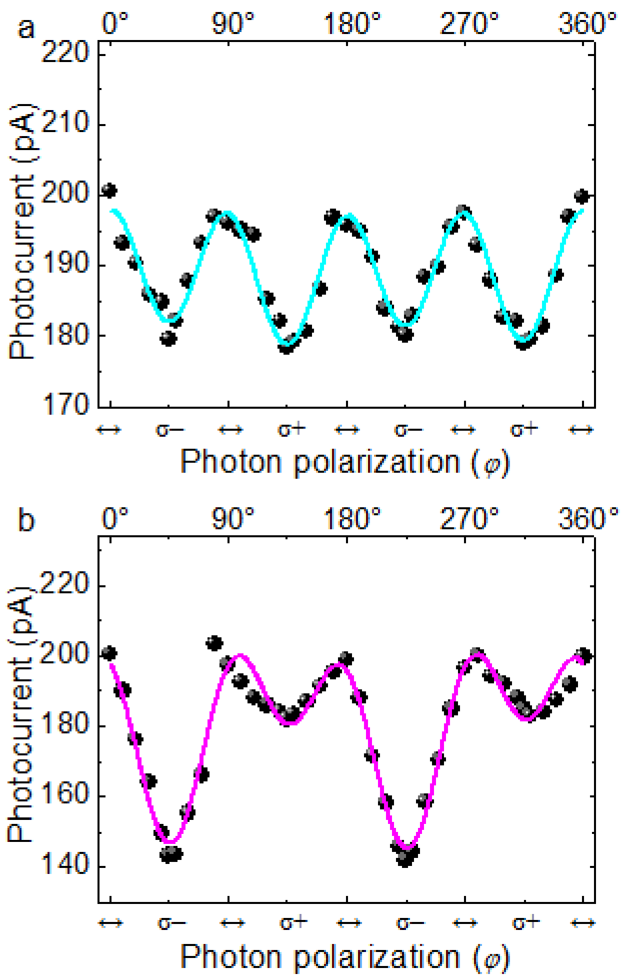

Supplementary Figure 14 Spin coupled valley dependent dichroic photocurrent in monolayer MoS_2 prepared by mechanical cleaving. a Photocurrent (PC) as a function of angle of photon polarization φ when illuminated by 2.33 eV (off-resonance with excitons) laser, for the monolayer MoS_2 sample prepared by mechanical exfoliation (S-MC). The (cyan) curve is the fitting function based on the phenomenological PC formula for monolayer MoS_2, yielding negligible polarization. **b** PC amplitude as a function of angle of incidence of polarization when illuminated by 1.96 eV (on-resonance with ***A*** exciton) laser, for S-MC. The (magenta) curve is the fitting function based on the phenomenological PC formula for monolayer MoS_2, yielding a polarization of ~ 40 ± 8 %. The data at 1.96 eV excitation show large PC variation and much clear helicity dependent PC which may be due to larger exciton density in S-MC.

	C_1	L_1	L_2	D
1.96 eV	-0.2	-1.1	5.4	146.7
2.33 eV	-0.3	0.3	4.1	107.0

Supplementary Table 1 | Photocurrent components of the monolayer MoS_2 grown by chemical vapor deposition, excited at normal incidence. Parameters are determined from the phenomenological photocurrent (PC) fittings for the monolayer MoS_2 grown by chemical vapor deposition (S-CVD). The PC components obtained by 1.96 and 2.33 eV circularly polarized excitations at θ = 0˚. For both on-resonance (1.96 eV) and off-resonance excitations, C_1 is negligibly small. As expected, the circular photogalvanic effect not observed for no oblique incidence (θ = 0˚, normal incidence). The linear photogalvanic L_1 and linear photon drag terms L_2 are the only observed polarization dependent components.

	C_1	L_1	L_2	D
1.96 eV	-17.5	1.7	16.5	180.2
2.33 eV	-1.3	0.1	8.6	188.9

Supplementary Table 2 | Photocurrent components of the monolayer MoS_2 sample prepared by mechanical cleaving excited by circularly polarized light. Parameters determined from the phenomenological photocurrent (PC) fittings of the monolayer MoS_2 sample prepared by mechanical cleaving (S-MC) for an oblique incidence of $\theta = 45^{\circ}$. The PC components obtained by 1.96 and 2.33 eV circularly polarized excitations.

Supplementary Note 1 | Helicity dependent photocurrent observed for excitation on-resonance with the excitonic transitions.

When we pump at 1.96 eV, we pump just above the ***A*** exciton but below the ***B*** exciton. This excitation is on resonance with either ***A*** or ***B*** excitons. However, it can populate mainly ***K*** valleys for σ+ excitations and –***K*** valleys for σ– excitations related to the ***A*** excitons[6]. Since ***A*** excitons are much stronger in intensity than the ***B*** excitons and 1.96 eV is an energy below the ***B*** excitons, populations in ***K*** (–***K***) valleys due to σ+ (σ–) excitations related to the ***B*** excitons are negligible.

Supplementary Note 2 | Helicity dependent photocurrent of monolayer MoS_2 and possible source of differences.

Helicity dependent photocurrent (PC) for off-resonance and on-resonance excitation of the monolayer MoS_2 prepared by mechanical cleaving (S-MC) is given in Supplementary Figure 14. PC parameters were extracted using the phenomenological fitting (green fitting in Supplementary Figure 14a and red fitting in Supplementary Figure 14b) and are shown in Supplementary Table 1. PC polarization is ~ 40 ± 8 %.

There could be several reasons for the fluctuations of the helicity dependent PC in the monolayer MoS_2 grown by chemical vapor deposition (S-CVD) shown in the main text. One straightforward reason could be due to the device fabrication, which can be enhanced and it is an ongoing work. If this is an intrinsic property, it could be due the fact that the density of excitons (main text, Figure 1) in S-CVD is much lower than that in mechanically cleaved (S-MC) samples. Indeed, well-defined helicity dependent PC response can be seen in S-MC in Supplementary Figure 14. Another note is about the nature of the intrinsic strain in S-CVD. The relatively smaller compressive strain may lead to a pronounced valley polarization[6] in S-CVD sample. The large fluctuations in S-CVD can be alternatively explained in terms of non-uniform emission-absorption processes in CVD samples. As a result, valley currents in different valleys can be affected by these non-uniformities, leading to a largely varying PC polarization, when excited by 1.96 eV on-resonance laser. On the other hand, when samples are excited by 2.33 eV laser, which is off-resonance for both ***A*** and ***B*** excitons, there are no fluctuations in valley current. The latter also implies the role of on-resonant excitation with excitons and its relation to the j_{CPGE} and PC polarization.

References

[1] Wang, Y. L., Cong C. X., Qiu C. Y. & Yu T. Raman spectroscopy study of lattice vibration and crystallographic orientation of monolayer MoS_2 under uniaxial strain. *Small* **9,** 2857-2861 (2013).

[2] Mak, K. F. *et al.* Tightly bound trions in monolayer MoS_2. *Nat. Mater.* **12,** 207-211 (2013).

[3] Lopez-Sanchez, O., Lembke, D., Kayci, M., Radenovic, A. & Kis, A. Ultrasensitive photodetectors based on monolayer MoS_2. *Nat. Nanotechnol.* **8,** 497-501 (2013).

[4] Finkelstein, G., Shtrikman, H. & Bar-Joseph, I. Negatively and positively charged excitons in GaAs/Al_xGa_{1-x}As quantum wells. *Phys. Rev. B* **53,** R1709–R1712 (1996).

[5] Lu, C.-P., Li, G., Mao, J., Wang, L.-M. & Andrei, E. Y. Bandgap, mid-gap states, and gating effects in MoS_2. *Nano Lett.* **14,** 4628-4633 (2014).

[6] Ji, Q. *et al.* Epitaxial monolayer MoS_2 on mica with novel photoluminescence. *Nano Lett.* **13,** 3870-3877 (2013).
